# Promoting Homogeneous Zinc‐Ion Transfer Through Preferential Ion Coordination Effect in Gel Electrolyte for Stable Zinc Metal Batteries

**DOI:** 10.1002/advs.202304915

**Published:** 2023-10-23

**Authors:** Sangyeop Lee, Im Kyung Han, Na Gyeong Jeon, Yubin Lee, Hye Bin Son, Dong‐Yeob Han, Seoha Nam, Taehun Chung, Myung‐Jun Kwak, Youn Soo Kim, Soojin Park

**Affiliations:** ^1^ Division of Advanced Materials Science Pohang University of Science and Technology (POSTECH) 77 Cheongam‐ro, Nam‐gu Pohang 37673 Republic of Korea; ^2^ Department of Materials Science and Engineering Pohang University of Science and Technology (POSTECH) 77 Cheongam‐ro, Nam‐gu Pohang 37673 Republic of Korea; ^3^ Department of Chemistry Pohang University of Science and Technology (POSTECH) 77 Cheongam‐ro, Nam‐gu Pohang 37673 Republic of Korea; ^4^ Advanced Batteries Research Center (ABRC) Korea Electronics Technology Institute (KETI) 25 Saenari‐ro, Bundang‐gu Seongnam 13509 Republic of Korea

**Keywords:** aqueous rechargeable batteries, ion coordination, preferential coordination, zinc metal anodes, zwitterionic gel electrolytes

## Abstract

Aqueous zinc metal batteries (AZMBs) are emerging energy storage systems that are poised to replace conventional lithium‐ion batteries owing to their intrinsic safety, facile manufacturing process, economic benefits, and superior ionic conductivity. However, the issues of inferior anode reversibility and dendritic plating during operation remain challenging for the practical use of AZMBs. Herein, a gel electrolyte based on zwitterionic poly(sulfobetaine methacrylate) (poly(SBMA)) dissolved with different concentrations of ZnSO_4_ is proposed. Two‐dimensional correlation spectroscopy based on Raman analysis reveals an enhanced interaction priority between the polar groups in SBMA and the dissolved ions as electrolyte concentration increases, which establishes a robust interaction and renders homogeneous ion distribution. Attributable to the modified coordination, zwitterionic gel polymer electrolyte with 5 mol kg^−1^ of ZnSO_4_ (ZGPE‐5) facilitates stable zinc deposition and improves anode reversibility. By taking advantage of preferential coordination, a symmetrical cell evaluation employing ZGPE‐5 demonstrates a cycle life over 3600 h, where ZGPE‐5 also exerts a beneficial effect on the full cell cycling when assembled with Zn_0.25_V_2_O_5_ cathode. This study elucidates changes in the internal ion behavior that are dependent on electrolyte concentrations and pave the way for durable AZMBs.

## Graphical abstract

A zwitterionic polymer‐based gel electrolyte is proposed for an advanced aqueous zinc metal battery. By controlling the concentration of dissolved salt, electrochemical behaviors can be modified, attributable to the change in the coordination priority order. Accordingly, the interaction between charge carriers and the gel matrix is intensified, which contributes to the improved reversibility of the zinc anode.

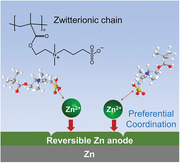

## 1 Introduction

As climate change has developed into a pressing global environmental concern, the demand for eco‐friendly electricity as an alternative to fossil fuels has grown increasingly urgent.^[^ ^1^, ^2^ ^]^ However, sustainable energy sources that harness natural resources for electricity generation frequently encounter issues of intermittency and instability.^[^ ^3^, ^4^ ^]^ Accordingly, electrochemical energy storage technologies like rechargeable batteries have gained prominence as a logical solution to these problems by storing generated energy.^[^ ^5^ ^]^ However, commercial lithium‐ion batteries (LIBs) remain prone to accidental explosions resulting from thermal runaway incidents due to their organic solvent‐based electrolyte, which can initiate aggressive exothermic reactions of organic solvents and a steep rise in internal temperature.^[^ ^6^ ^]^ Thus, eliminating the potential risk of fire to prevent safety accidents caused by conventional LIBs has emerged as a significant issue in the field of battery research.^[^ ^7^, ^8^ ^]^

Alternatively, water‐based aqueous zinc (Zn) metal batteries (AZMBs) that employ Zn metal and Zn^2+^ ions as an anode material and charge transfer carrier, respectively, offer inherent safety and additional advantages such as high ionic conductivity, economic benefits, eco‐friendliness, high energy density (5854 mAh cm^−3^), and natural compatibility with water.^[^ ^9^, ^10^, ^11^, ^12^, ^13^, ^14^ ^]^ However, the Zn anode undergoes severe damage due to dendritic growth during charge/discharge cycles, posing a significant hurdle to the commercialization of AZMBs.^[^ ^15^ ^]^ As with other metal anodes, an uneven distribution of Zn^2+^ ions and Zn atom surface diffusion results in the formation of metal dendrites, leading to impaired electrode reversibility and potential internal short circuits.^[^ ^16^ ^]^ Thus, the limited cyclability stemming from Zn dendrites makes the suppression of dendrite formation a critical research focus for AZMBs. To overcome this obstacle, researchers have explored various approaches, such as introducing versatile layers,^[^ ^17^, ^18^ ^]^ novel electrolyte design,^[^ ^19^, ^20^ ^]^ electrode structure engineering,^[^ ^21^ ^]^ anode modifications,^[^ ^22^ ^]^ functional separator,^[^ ^23^ ^]^ and polymer‐based electrolytes.^[^ ^24^ ^]^

Particularly, gel polymer electrolyte (GPE) engineering holds considerable potential among various strategies as it is closely linked to the migration of internal ions and corresponding electrodeposition behavior. GPEs also possess the advantage of providing unique mechanical and electrochemical characteristics based on the polymer matrix utilized.^[^ ^25^ ^]^ For instance, it is suggested that zwitterionic polymers which comprise both cationic and anionic groups simultaneously along chains allow multiple functional groups to initiate intensive interactions with electrolyte species, where anionic polar functional groups within the polymer chain interact readily with dissolved Zn^2+^ ions, facilitating the formation of an ion transport pathway.^[^ ^26^, ^27^ ^]^ The electrostatic force between the functional groups and Zn^2+^ results in the alignment of Zn^2+^ along the polymer chain, which acts as a guide for cation transport under an electric field, thereby serving as an ion transfer channel.^[^ ^28^ ^]^ The resulting pathway averts the ion concentration gradient and the growth of mossy Zn structures, which leads to uniform and in‐plane metal electrodeposition.^[^ ^29^ ^]^ Intriguingly, the concentration of dissolved salt affects the coordination behavior of polar groups and rearranges the interaction priority order between the polymers and internal ions (i.e., promoting preferential coordination). Additionally, the induced change in the internal environment not only offers modified metal plating behaviors but also enhances the electrode cycle life based on improved anode reversibility. Adopting zwitterionic polymers in GPEs has been reported to enhance electrode stability; however, research on the effects of varying electrolyte concentrations on coordination priority order and electrochemical behavior is limited.^[^ ^30^, ^31^, ^32^ ^]^

**Figure 1:**
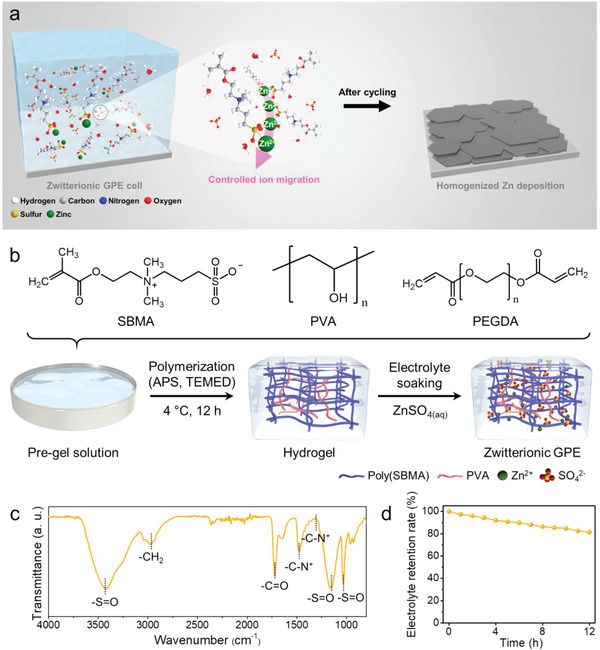
Working mechanism and characterization of the ZGPE. a) Schematic illustration of the zwitterionic gel electrolyte for Zn metal batteries. b) Synthesis procedure of the ZGPE from the zwitterionic repeating unit and polymeric materials, undergoing polymerization of pre‐gel solution and immersion in ZnSO_4_ aqueous solution sequentially. c) FT‐IR spectra of the fabricated zwitterionic hydrogel before electrolyte soaking. d) Electrolyte retention rate of ZGPE‐5 over time at room temperature under a relative humidity of 60%.

Herein, we present sulfobetaine methacrylate (SBMA) as a repeating unit for the zwitterionic polymer matrix and examine the improvement of anode reversibility through preferential ion coordination behavior. The abundant polar groups in SBMA interact favorably with internal ions and influence charge carrier migration during cell operation. Using two‐dimensional (2D) correlation spectroscopy in conjunction with Raman spectroscopy, it has been found that the interaction priority order of SO_3_^−^ functional groups increases when the concentration of ZnSO_4_ salt in the gel electrolyte rises from 2 m (mol kg^−1^) to 5 m. Preferential coordination stimulates the uniform distribution of Zn^2+^ ions within the structure and alleviates the tip effect, contributing to improved stability during electrodeposition. Additionally, the combined effect of a zwitterionic gel and an electrostatic interaction hinders the 2D diffusion of Zn atoms on the anode surface and enables a dendrite‐free electrode morphology accordingly (**Figure** **1**a). As a result, a zwitterionic gel polymer electrolyte (ZGPE) with 5 m ZnSO_4_ supports durable aqueous Zn metal batteries through uniform Zn plating/stripping behavior, as demonstrated by long‐term symmetrical cell results (>3600 h cycle life at an areal capacity of 0.5 mAh cm^−2^ and a current density of 0.5 mA cm^−2^). The feasibility of the proposed system was further validated by Zn|Zn_0.25_V_2_O_5_ full cells, which exhibited excellent cyclability as preferential ion coordination retains the anode sustainability and prevents performance degradation.

## 2 Results and Discussion

### 2.1 Fabrication Process and Characterization of ZGPE

Figure 1b provides a schematic representation of the overall fabrication procedure for a ZGPE, highlighting the selection of zwitterionic SBMA as a rational gel precursor due to its concurrent presence of cationic quaternary ammonium and anionic sulfonate functionalities. Briefly, ZGPE was prepared by radical polymerization of SBMA, and poly(ethylene glycol) diacrylate (PEGDA) was simultaneously adopted as a crosslinker to develop a sturdy polymer matrix. Additionally, a small quantity of poly(vinyl alcohol) (PVA) was dissolved in distilled water and formed an aqueous mixture of SBMA, PEGDA, PVA, and initiator. The pre‐gel solution was subsequently transferred to a mold with a target thickness of 500 µm to synthesize a hydrogel based on zwitterionic polymer chains. The fabricated hydrogel, which is composed of a semi‐interpenetrating polymer network (semi‐IPN) structure, featuring polymerized SBMA (poly(SBMA)) and PVA chains, was immersed in liquid electrolytes with different ZnSO_4_ concentrations (2 and 5 m of ZnSO_4_ aqueous solutions) to obtain transparent ZGPE (Figure S1, Supporting Information).

The chemical composition of the polymer matrix without electrolyte species was analyzed in depth through Fourier transform infrared spectroscopy (FTIR) in Figure 1c. The FTIR results reveal a well‐developed polymer matrix, displaying intrinsic transmittance peaks of the SBMA unit and PVA chain after polymerization (Figure S2a,b, Supporting Information). The intense transmittance peak at 3415 cm^−1^ corresponds to the ─OH bond originating from the components in a hydrogel, while the distinctive peak at 2970 cm^−1^ indicates the C─H stretching in the incorporated polymeric chains.^[^ ^33^, ^34^ ^]^ The sharp peak at 1720 cm^−1^ is attributed to the ester C═O stretching present in the SBMA unit and PEGDA crosslinker.^[^ ^35^ ^]^ Furthermore, two characteristic transmittance peaks at 1170 and 1030 cm^−1^ signify asymmetrical and symmetrical stretching of S═O, respectively, which are related to the sulfonate groups in SBMA units.^[^ ^36^ ^]^ Also, the peaks at 1301 and 1472 cm^−1^ are assigned to the C─N^+^ bond of quaternary ammonium in SBMA, illustrating the effective integration of both cationic and anionic functionalities.^[^ ^30^, ^37^ ^]^ It is noteworthy that the peaks appearing ≈850 cm^−1^ in the SBMA monomer, which originates from the vibrational peak of C═C, are not present after the polymerization into poly(SBMA) during the formation of the hydrogel matrix, indicating the successful fabrication of a uniform and stable structure.

PVA was selected owing to its exceptional hydrogen bonding capabilities, anticipated to enhance the mechanical properties of ZGPE. To validate the superior physical properties of the hydrogel, a weight of 1 kg was placed on the zwitterionic hydrogel, and consequential structural changes were observed (Figure S3, Supporting information). Notably, even after being subjected to significant pressure, the hydrogel maintained its original structure, which underscores the exceptional toughness of the proposed gel system, suggesting its potential as a gel electrolyte resilient against external pressures like cell assembly and Zn volume expansion. Furthermore, the reinforcement effect is likely to be intensified due to augmented hydrogen bonding among PVA chains, attributed to the “salting out” effect derived from SO_4_^2−^ ions in the electrolyte as described by the Hofmeister anion series.^[^ ^38^ ^]^ Owing to the enhanced “salting out” effect in the increased concentration, the ZGPE with 5 m ZnSO_4_ (termed as ZGPE‐5) exhibited higher Young's modulus value (*E*) of 58 kPa compared to the ZGPE with 2 m ZnSO_4_ (termed as ZGPE‐2) presenting *E* of 15 kPa (Figure S4, Supporting Information). Meanwhile, the meticulously structured polymer matrix is expected to endow the ZGPE with excellent water retention due to the cooperative effect of sufficient charge groups and the strong hydrogen bonding interaction between water molecules and PVA chains. To quantify the extent of water uptake, a ZGPE impregnated with 5 m ZnSO_4_ solution was left in a chamber with a relative humidity of 60%, and the ensuing change in electrolyte retention was examined (Figure 1d). As a result, ZGPE‐5 exhibited an impressive retention rate of 81% compared to its initial moisture even after 12 h in an open system indicative of its remarkable electrolyte retention function. In addition, when compared to the pristine hydrogel and ZGPE‐2, ZGPE‐5 demonstrated superior electrolyte retake under diverse relative humidity conditions, evidencing its potential for application in gel electrolytes (Figure S5a,b, Supporting Information). To further elucidate the characteristics of the proposed gel system, the internal structures of the hydrogel, ZGPE‐2, and ZGPE‐5 were observed in the equilibrium swollen state using confocal laser scanning microscopy (CLSM). Prior to the CLSM measurement, respective gels were immersed at 25 °C for 2 days in an aqueous solution of a fluorescent probe enabling the hydrophobic domain of hydrogels to be identified.^[^ ^39^, ^40^ ^]^ Figure S6 (Supporting Information) reveals that the fabricated hydrogels, regardless of the electrolyte concentration, demonstrated homogeneous fluorescent intensity over all ranges due to the absence of hydrophobic domains, indicating that the polymer chains can interact readily with dissolved Zn^2+^ ions.

### 2.2 Effect and Working Mechanism of ZGPE Concentration Modulation

**Figure 2:**
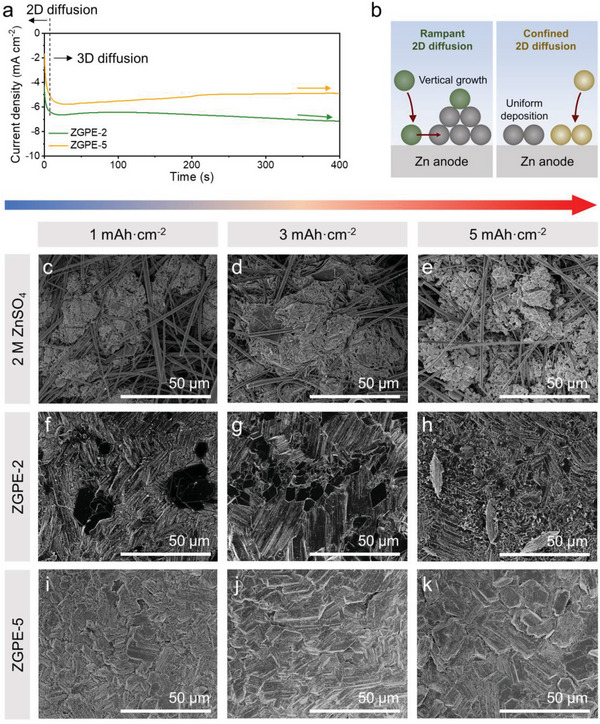
Effect of the gel concentration on electrochemical behavior. a) Chronoamperometry results of cells employing ZGPE‐2 and ZGPE‐5. b) Schematic description for atomic diffusion behavior on Zn anode surface indicating vertical growth and uniform deposition of Zn, respectively. Top‐view SEM images of deposited Zn electrodes using 2 m ZnSO_4_ liquid electrolyte under the areal capacity of c) 1, d) 3, and e) 5 mAh cm^−2^. Top‐view SEM images of deposited Zn electrodes using ZGPE‐2 under the areal capacity of f) 1, g) 3, and h) 5 mAh cm^−2^. Top‐view SEM images of deposited Zn electrodes using ZGPE‐5 under the areal capacity of i) 1, j) 3, and k) 5 mAh cm^−2^.

The suitability of the fabricated ZGPE for an aqueous battery system was briefly assessed by measuring the ionic conductivity of the ZGPE with different concentrations. Typically, increased solution viscosity and the resulting slow ion migration often lead to lower ionic conductivity in concentrated electrolytes.^[^ ^41^ ^]^ However, when combined with the zwitterionic matrix, the concentrated ZGPE‐5 maintained a comparable ionic conductivity of 21.6 mS cm^−1^ to that of ZGPE‐2 (24.6 mS cm^−1^) (Figure S7, Supporting Information). The extraordinary ion‐conducting capability of ZGPE is attributed to the beneficial properties of the incorporated zwitterionic polymers, which facilitate salt dissociation and enhance internal ion transport.^[^ ^42^ ^]^ To further probe the potential of ZGPE for AZMBs, an electrochemical analysis using chronoamperometry was performed, aiming to determine the direction of Zn metal growth on the anode surface during the initial stages of the electrodeposition. In the graph of time and current density under constant bias, an increase in current during the early phase signifies the nucleation and 2D diffusion of Zn^0^ atoms on the electrode surface as part of the electrodeposition process, while the latter region represents a 3D translation of Zn^0^ atoms.^[^ ^43^ ^]^ Noticeably, the cells with 2 m (mol/L) ZnSO_4_ liquid electrolyte showed a consistent increase in current, inferring an unceasing vertical Zn deposition due to unequal charge distribution and inability to limit surface diffusion (Figure S8, Supporting Information). Conversely, ZGPE‐based cells demonstrated diminished atomic diffusion compared to liquid electrolyte cells (**Figure** **2**a). Intriguingly, in the ZGPE‐2‐based cell, Zn progressively accumulated in an out‐of‐plane direction over time due to the rampant surface diffusion of Zn^0^ toward energetically favorable sites, whereas the current in the ZGPE‐5 remained stable indicating successful inhibition of dendritic Zn growth during continuous plating (Figure 2b). The discrepancy in plating behavior is supposed to stem from the distinct ion guiding effects between ZGPE‐2 and ZGPE‐5 that the level of ability to control internal ions depends on the electrolyte concentration.

To visually explore the effect of ZGPE on cell operation, Zn|Zn symmetric cells with different electrolytes (2 M ZnSO_4_ liquid electrolyte, ZGPE‐2, and ZGPE‐5) were fabricated and Zn was sequentially plated on each working electrode for 1, 3, and 5 mAh cm^−2^ at a constant current density of 0.5 mA cm^−2^. The morphological changes in the anode were examined using a top‐view scanning electron microscope (SEM) analysis and it is noteworthy that a gentle cleaning process was carried out before the observation to remove the remaining electrolyte species. The pristine Zn foil displayed a smooth and clean surface before applying current, minimizing the influence of the intrinsic roughness of the metal foil on electrochemical behavior (Figure S9, Supporting Information). In the case of the liquid electrolyte, randomly oriented metal growth caused entanglement between glass fiber strands and Zn flakes regardless of capacity, which is suspected to negatively affect cycling behavior (Figure 2c–e). On the other hand, electrodes integrated with ZGPE display horizontally evolved crystal structures, which correspond with the hexagonal close‐packed structure of Zn crystals.^[^ ^44^ ^]^ However, a degree of surface diffusion in ZGPE‐2 led Zn atoms to cluster at certain points, causing uneven protrusions in electrodes with 1 and 3 mAh cm^−2^ of Zn deposited (Figure 2f,g). This disparity becomes more pronounced in the cell electrodeposited at 5 mAh cm^−2^ and multiple sizeable aggregates are distinctly noticeable (Figure 2h). Unfortunately, the vertically grown crystals can potentially trigger internal short circuits, a consequence due to the inability of ZGPE‐2 to entirely manage the distribution of internal ions. Instead, ZGPE‐5 exhibits a homogeneous and planar electrode morphology due to its superior ion allocation ability and diffusion‐suppressing effect (Figure 2i,j). Importantly, even when dealing with the comparatively high capacity of 5 mAh cm^−2^, the overall structure was still uniformly formed into hexagonal shapes (Figure 2k). Also, it should be noted that the plating test using 2 and 5 m liquid electrolytes revealed no distinct differences between the two electrodes (Figure S10a,b, Supporting Information).

Additionally, variations in plating behavior depending on the type of electrolyte were elucidated through X‐ray diffraction (XRD) analysis. The peak intensity ratio between (002) and (100), referred to as I_(002)_/I_(100)_, serves as an indicator for Zn growth direction and provides insights into the uniformity of Zn deposition as well as the extent of dendrite formation during plating.^[^ ^45^ ^]^ By employing ZGPE‐2 and ZGPE‐5 as electrolytes and increasing deposition capacity from 1 to 5 mAh cm^−2^, XRD analysis revealed the influences of the electrolytes on the directional growth of Zn during continuous plating (Figure S11a,b, Supporting Information). The analysis results indicated no discernible growth pattern in *I*_(002)_/*I*_(100)_ values in cells using ZGPE‐2, thereby suggesting random plating sequences. Conversely, the cells with ZGPE‐5 displayed an initially low *I*_(002)_/*I*_(100)_ ratio that gradually increased, offering evidence that Zn could grow in a uniform direction as the plating process continued. Thus, the electrochemical property of ZGPE‐5 is anticipated to prevent stagnation in charge transfer and the occurrence of short circuits caused by dendritic growth, thereby contributing to improved cell cycling. Furthermore, alongside the enhanced deposition stability with ZGPE‐5, cyclic voltammetry (CV) results showed improved reversibility in plating/stripping behavior when using ZGPE‐5 (Figure S12, Supporting Information). In the CV analysis carried out with ZGPE‐2 and ZGPE‐5, the cell utilizing ZGPE‐5 exhibited a reduced gap between cathodic and anodic peak potentials, coupled with an increased area under the anodic peak.^[^ ^46^ ^]^

Differences in the electrochemical behaviors of ZGPE‐2 and ZGPE‐5 during the process of metal reduction are hypothesized to arise from variances in the interactions between ions dissolved in the electrolyte and the zwitterionic polymer matrix. To estimate the changes in properties of ZGPE depending on electrolyte concentration, FTIR analysis was conducted using a zwitterionic hydrogel, ZGPE‐2, ZGPE‐5, and aqueous solutions with 2 and 5 m ZnSO_4_, respectively (Figure S13, Supporting Information). The characteristic peaks at 1030 and 1170 cm^−1^ in the spectra represent vibrations due to S═O bonds in the sulfonate of SBMA, while the peak at 1085 cm^−1^ indicates S═O vibration in the sulfate of ZnSO_4_ salt.^[^ ^47^ ^]^ Interestingly, ZGPE showed a blueshift compared to an aqueous solution of the same concentration due to the suppressed interaction between sulfate and Zn^2+^ affected by the polymer‐Zn^2+^ interaction, which indicates that Zn^2+^ ions can favorably coordinate with the polymer matrix. Concurrently, an increase in electrolyte concentration from 2 to 5 m affects the number of S═O bonds in poly(SBMA) displaying an asymmetrical vibration mode, resulting in a reduced intensity of the sulfonate peak at 1170 cm^−1^.^[^ ^48^ ^]^ This occurs due to the intensified interaction between Zn^2+^ and sulfonate with increasing electrolyte concentration, thereby supporting the distinct behavior of ZGPE under different concentrations.

**Figure 3:**
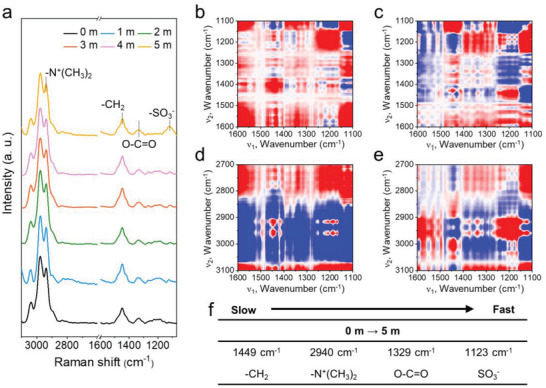
Investigation on the working mechanism of ZGPE‐5. a) 1D Raman analysis results of ZGPE with increasing salt concentration from 0 to 5 m, featuring positions between 1100 and 3100 cm^−1^. b,d) Synchronous and c,e) asynchronous 2D Raman correlation spectra of ZGPE during increasing salt concentration 0–5 m. f) The sequential order of spectral changes of ZGPE as revealed by Raman analysis with increasing salt concentration from 0 to 5 m.

To further affirm that the properties of ZGPE change according to the concentration of electrolyte dissolved within the gel, Raman spectra of electrolyte‐equilibrated ZGPE were obtained ranging from 0 to 5 m of ZnSO_4_ (**Figure** **3**a). The characteristic peak at 1123 cm^−1^ is indicative of ‐SO_3_^−^ vibrations by the anionic sulfonate groups contained in the zwitterionic unit, while a broad peak found at 1329 cm^−1^ signifies the O─C═O bond.^[^ ^49^ ^]^ Additionally, the peak at 2940 cm^−1^ represents cationic tertiary ammonium bonds (─N^+^(CH_3_)_2_) originating from the SBMA repeating unit.^[^ ^50^ ^]^ Meanwhile, the ─CH_2_ bond, which is widely distributed throughout the polymer structure, offers a sharp peak at 1449 cm^−1^.^[^ ^51^ ^]^ We also note that S─O peaks originating from ZnSO_4_ are excluded from this analysis because they present in regions below 1000 cm^−1^, a range that poses challenges for precise analysis due to limitations inherent to the measurement of samples employing hydrogels.^[^ ^52^ ^]^ Based on the Raman analysis, 2D correlation spectroscopy (2D COS) analysis was implemented to deeply understand the intermolecular interactions and elucidate the sequential order of interactions among functional groups in the polymer chain and electrolyte species resulting from changes in electrolyte concentration.^[^ ^53^ ^]^ In the synchronous Raman 2D correlation spectrum, red auto peaks signify correlation peaks changing in the same direction with increasing electrolyte concentration (i.e., either increasing or decreasing with rising concentration of electrolytes), while blue cross‐peaks represent correlation peaks changing in opposite directions. According to Noda's law,^[^ ^54^, ^55^ ^]^ if the correlated peaks in the asynchronous Raman 2D spectra display the same color and position as in the synchronous spectra, the peak at the *x*‐axis changes due to concentration prior to the y‐axis peak. On the other hand, if the colors vary, concentration differences trigger changes in the *y*‐axis peak before the x‐axis peak. Accordingly, considering both signs of the synchronous and asynchronous spectra, a clear preferential response is demonstrated by carbonyl (O─C═O) groups as salt concentration increased from 0 to 2 m (Figures S14a–d and S15, and Table S1, Supporting Information). The analysis result suggests that, in the case of ZGPE‐2, the absence of primary coordination between polar groups in SBMA and internal ions results in Zn^2+^ charge carriers remaining in a weakly coordinated state, hindering the designed role of the zwitterionic gel in guiding ion migration. The deficient ion guiding effect potentially causes an ion concentration gradient during the electroplating process, promoting the dendritic growth of Zn. Conversely, ZGPE with a salt concentration increased to 5 m exhibits a substantially different trend in the sequential order compared to preceding results with lower salt concentrations (Figure 3b–e; Table S2, Supporting Information). As a result of the 2D COS analysis, it becomes evident that the distinctive functional groups ‐SO_3_^−^ within the zwitterionic SBMA exhibited a preferential interaction than other functional groups as salt concentration increased from 0 to 5 m (Figure 3f). Importantly, the modified interaction order of the sulfonate group significantly affects the electrochemical properties as the negatively charged ‐SO_3_^−^ promotes robust interaction with Zn^2+^ ions, which mitigates the uneven distribution of Zn^2+^ ions across the entire gel structure.^[^ ^56^, ^57^ ^]^ Thus, when ZGPE‐5 is employed, the priority order of interactions between the polymer chains and internal ions changes and facilitates the formation of a Zn^2+^ ion transfer pathway. Consequently, the ZGPE‐2 demonstrates insufficient modification of electrochemical properties, as internal ions primarily interacted with functional groups other than the sulfonate group of SBMA. On the other hand, in the case of ZGPE‐5, the sulfonate groups preferentially interact with Zn^2+^ ions, leading to improved electrochemical properties by effectively preventing an ion concentration gradient during the charge/discharge process. Furthermore, upon evaluating the transference number of Zn^2+^ (t_zn2+_) to determine the contribution of Zn^2+^ among the entire charge carriers, the measured results revealed that a cell utilizing ZGPE‐2 demonstrated a relatively low value of 0.61 (Figure S16a, Supporting Information). On the other hand, the ZGPE‐5 showed a t_zn2+_ of 0.86 due to the strong coordination between Zn^2+^ ions and the polymer framework (Figure S16b, Supporting Information).

### 2.3 Electrochemical Performance of ZGPE‐Based Cells

**Figure 4:**
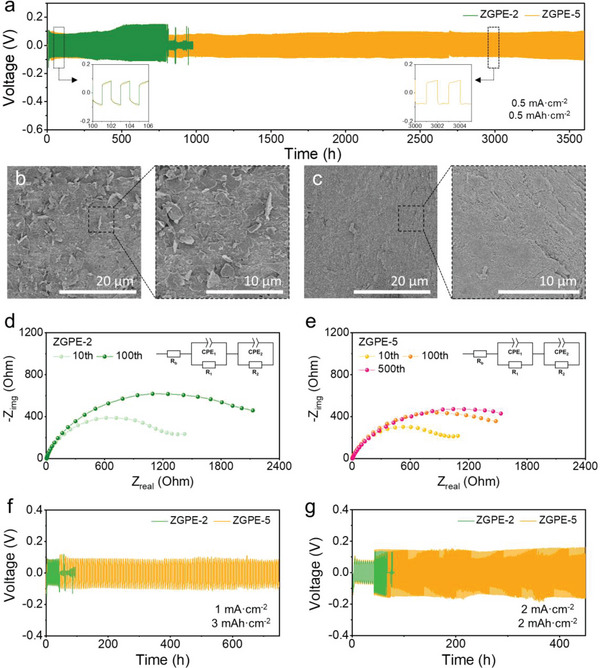
ZGPE‐based Zn symmetric cell investigation. a) Long‐term symmetric cell cycling performance for cells using ZGPE‐2 and ZGPE‐5 under an areal capacity of 0.5 mAh cm^−2^ and a current density of 0.5 mA cm^−2^. Top‐view SEM images of Zn anode after 100 repetitive plating/stripping processes with b) ZGPE‐2 and c) ZGPE‐5. Nyquist plots of cells employing d) ZGPE‐2 and e) ZGPE‐5 at the 10th and 100th cycles, respectively, where the ZGPE‐5 further shows the impedance analysis result of the 500th cycle. Inset figures illustrate the equivalent circuit model of the proposed system. Symmetrical cell cycling results for ZGPE‐2 and ZGPE‐5 based cells f) under an areal capacity of 3 mAh cm^−2^ and a current density of 1 mA cm^−2^, and g) under an areal capacity of 2 mAh cm^−2^ and a current density of 2 mA cm^−2^.

The preferential interaction of the abundant polar groups in the zwitterionic matrix with Zn^2+^ ions leads to the expectation of enhanced cycling properties for Zn metal batteries paired with ZGPE‐5. To verify beneficial effects, Zn|Zn symmetrical coin cells containing different electrolytes were constructed and their electrochemical performance was analyzed. First, long‐term cyclability was tested at an areal capacity of 0.5 mAh cm^−2^ under a constant current density of 0.5 mA cm^−2^ to identify anode reversibility. As illustrated in Figure S17 (Supporting Information), the cell based on the 2 M ZnSO_4_ liquid electrolyte encountered abrupt cell failure due to an internal short circuit. Importantly, intense charge concentration causes an imbalance in Zn deposition and promotes the formation of vertically grown Zn dendrites in liquid electrolytes.^[^ ^58^ ^]^ Conversely, ZGPE‐based cells display improved cycling behavior compared with liquid electrolytes that both ZGPE‐2 and ZGPE‐5 present stable performance in the initial operating phase (**Figure** **4**a). However, during the initial electrodeposition phase, the ZGPE‐5 displayed reduced nucleation overpotential and migration barrier values, which indicates that the ZGPE‐5 offers enhanced affinity with the Zn electrode and plays a pivotal role in promoting charge transfer (Figure S18, Supporting Information). Accordingly, the sporadic growth of Zn in ZGPE‐2 obstructs charge transfer between the electrolyte and electrode, resulting in a progressive increase in overpotential throughout the cycle and accelerated cell failure.^[^ ^59^, ^60^ ^]^ In contrast, the altered interaction order in ZGPE‐5 leads to a preferential transport of Zn^2+^ ions, which ensures a uniform distribution of internal ions and facilitates stable metal plating. The ZGPE‐5 consistently shows stable voltage hysteresis without internal short circuits even after 1500 repetitive plating/stripping processes, highlighting the excellent electrochemical reversibility of the ZGPE‐5 paired Zn anode. Consequently, ZGPE‐5 lasts for over 3600 h in symmetrical cell investigation, which is comparable to recent exemplary studies using gel electrolytes (Table S3, Supporting Information). Remarkably, no significant difference was identified when evaluating symmetrical cells with 2 and 5 m ZnSO_4_ liquid electrolytes, indicating that the superior cycling performance of ZGPE‐5 is a result of the introduced zwitterionic polymer gel (Figure S19a,b, Supporting Information).

A further investigation was carried out to determine the differences in cycling stability based on post‐mortem SEM analysis using Zn electrodes after 100 cycles under identical test conditions. Figure 4b reveals that concentrated charge migration results in an uneven surface on the ZGPE‐2‐based electrode after 100 cycles. Changes in surface morphology hinder the facile transfer of charge carriers and enlarge the surface area, thereby triggering electrochemical side reactions, which result in increased internal resistance.^[^ ^61^ ^]^ Additionally, such changes stimulate vertical deposition and cause internal shorts where two electrodes get interconnected.^[^ ^62^ ^]^ On the contrary, the ZGPE‐5 paired electrode maintained uniformity and largely restricted vertical Zn growth as shown in Figure 4c, indicating dendrite‐free anode over cycles. Electrochemical impedance spectroscopy (EIS) measurements were conducted after the 10th and 100th cycle to estimate the additional effect of salt concentration on electrochemical performances. In Figure 4d, the Nyquist plot indicates that the cell based on ZGPE‐2 shows low resistance after the 10th cycle, while the impedance value rapidly increases after the subsequent 100th cycle. The emergence of Zn dendrites widens the space between the gel and the electrode, hampering effective charge transfer and leading to a rise in internal resistance.^[^ ^63^ ^]^ Moreover, the dendritic surface elevates the number of sites for electrochemical reactions with electrolytes, thereby promoting detrimental side reactions that lead to the indispensable consumption of the electrolyte components causing severe depletion over the cycles.^[^ ^64^ ^]^ Alternatively, the electrode utilizing ZGPE‐5 exhibits a moderate rise in impedance over successive cycles, with the rate of increase diminishing as more cycles were completed (Figure 4e). Impressively, the resistance value after 500 cycles remained comparable to that observed after 100 cycles, which suggests that initial morphology variation induces an increase in resistance at the early phase, while stabilization in electrode transformation prevents further increment in impedance. The change in the electrode morphology that develops laterally throughout the plating/stripping processes effectively prevents the expansion of the gap between the electrolyte and the electrode as cycles continue, consequently supporting the maintenance of stable electrochemical properties.

Notably, the ZGPE‐5 exhibits improved cyclability even under elevated current density and areal capacity conditions. When subjected to a symmetrical cell test with an areal capacity of 3 mAh cm^−2^ under a current density of 1 mA cm^−2^ after stabilization cycling steps under mild test conditions, the ZGPE‐5‐based cell boasts a cycle life exceeding 700 h, whereas cells paired with ZGPE‐2 and 2 m ZnSO_4_ liquid electrolyte undergo early internal short circuit (Figure 4f; Figure S20, Supporting Information). Furthermore, electrodes employing ZGPE‐2 and 2 m ZnSO_4_ liquid electrolyte suffer from a rapid cell failure following the stabilization step attributed to excessive dendritic growth in a cyclability investigation at a higher current density of 2 mA cm^−2^ (Figure 4g; Figure S21, Supporting Information). Conversely, the cell incorporating ZGPE‐5 still performs enhanced cycling properties attributable to the preferential coordination effect, coupled with a negligible increment in overpotential throughout the cycles. Consequently, the enhanced stability of Zn electrodeposition, owing to the intensified interaction between the zwitterionic polymer chain and the charge carriers in the electrolyte, constructively supports the betterment of anode reversibility through the cycles and promotes the protracted lifespan of AZMBs.

**Figure 5:**
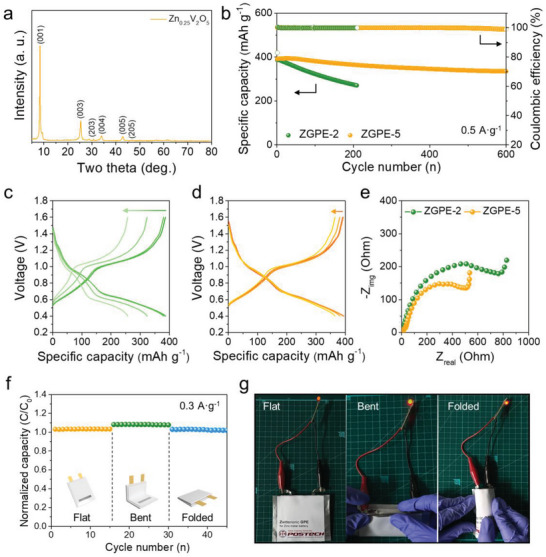
Electrochemical characterization of Zn|ZVO cells. A) XRD pattern of Zn_0.25_V_2_O_5_ cathode active material. B) Full cell cycling performance of ZGPE‐2 and ZGP‐5 based cells at a current density of 0.5 mA g^−1^. Voltage profiles of c) ZGPE‐2 and d) ZGPE‐5 for the 1st, 10th, 100th, and 200th cycle, respectively. e) EIS analysis results of Zn|ZVO cells with ZGPE‐2 and ZGPE‐5 as an electrolyte after 200 repetitive cycles. f) Change in the normalized capacity (*C*/*C*_0_, where *C*_0_ indicates an initial capacity) of assembled pouch cells under different mechanical deformations (flat, 90° bent, and 180° folded). g) Digital microscope images of cells connected in series to light an LED bulb operated in different mechanical states.

Based on the enhancement of anode reversibility in ZGPE‐5, a full cell performance evaluation was conducted by pairing the Zn anode with the Zn_0.25_V_2_O_5_ (ZVO) cathode to validate the feasibility for practical applications. The ZVO cathode active material was prepared by modifying previously reported methods, with detailed experimental procedures provided in the experimental section. The X‐ray diffraction pattern in **Figure** **5**a confirms the presence of characteristic peaks at 8.4°, 25.4°, 30.7°, 34.1°,43.1°, and 46.7° that represent (001), (003), (203), (004), (005), and (205) planes of ZVO, respectively, signifying the successful synthesis of crystalline cathode material.^[^ ^65^ ^]^ Furthermore, transmission electron microscope (TEM) analysis exposes an aligned interior lattice configuration of the nanorod‐like ZVO which provides the potential sites for charge storage (Figure S22a,b, Supporting Information).

A long‐term cyclability test was performed on Zn|ZVO full cells using ZGPE‐2 and ZGPE‐5 based on galvanostatic charge‐discharge investigation at a current density of 0.5 A g^−1^ (Figure 5b). Initially, both cells demonstrate stable capacity retention; however, as cycles progress, a distinct trend of decreasing charge capacity is observed in the cell with ZGPE‐2 due to anode degradation, showing ≈80% of the initial capacity (311 mAh g^−1^) only after 120 cycles. On the other hand, the cell employing ZGPE‐5 exhibits a mitigated capacity decrease induced by electrode deterioration. Accordingly, the ZGPE‐5 paired cell retains ≈96% of the initial capacity after 120 cycles with a charge capacity of 378 mAh g^−1^. Additionally, it shows enduring capacity retention throughout further cycling, maintaining ≈85% of the initial capacity even after 600 cycles. To scrutinize the degradation of electrode capacity per cycle more precisely, the voltage profile for the respective cycle was assessed. Figure 5c presents the voltage profiles concerning cell cycles for the ZGPE‐2‐based cell, which reveals a capacity of 387 mAh g^−1^ after the initial charge/discharge process. However, capacity decay becomes accelerated, showing charge capacities of 380, 323, and 257 mAh g^−1^ at the 10th, 100th, and 200th cycle, respectively, under identical current density, which represent capacity retentions of ≈98%, 83%, and 66% compared to the initial capacity. Conversely, ZGPE‐5 shows a marginally increased initial capacity of 392 mAh g^−1^ in the first cycle, suggesting enhanced stability for Zn^2+^ accommodation in the anode (Figure 5d). Moreover, the capacity decline is successfully restrained, leading to a remarkable capacity retention of ≈100%, 98%, and 94% after the 10th, 100th, and 200th cycles (393, 381, and 364 mAh g^−1^, respectively). The electrochemical stability of the cell using ZGPE‐5 was further evaluated via EIS analysis. Before cycling, Zn|ZVO cells using the two distinct types of electrolytes displayed similar impedance levels (Figure S23a, Supporting Information). Upon further evaluation after an additional 10 cycles, a modest increment is observed when compared to the pristine electrode, with this resistance increase noticeably presented in the cell using ZGPE‐2 (Figure S23b, Supporting Information). Moreover, the Nyquist plot analysis after 200 cycles further affirms the stability of ZGPE‐5, that the cell employing ZGPE‐5 demonstrates much lower charge transfer resistance compared to ZGPE‐2 suggesting stable ion migration and efficacy in mitigating electrode degradation (Figure 5e). Also, it is worth noting that when comparing the capacity retention of ZGPE‐2 and ZGPE‐5 employing cells as a function of increasing current density through rate capability investigation, no pronounced disparity was observed in electrochemical behaviors with varying current densities (Figure S24, Supporting Information). However, when the cell was subjected to a current density of 0.5 A g^−1^ for evaluating capacity recovery and cyclability after the rate test, the ZGPE‐2 demonstrated continuous capacity decay, attributed to cumulative anode deterioration during operation under high current density conditions. In contrast, while the ZGPE‐5 did not manifest improved electrochemical performance with elevated current densities, the ZGPE‐5 effectively curtailed damage to the anode, offering a positive contribution toward stability recovery after the rate test.

Meanwhile, water‐based electrolytes hold a high degree of compatibility regarding safety which is a vital consideration for wearable devices.^[^ ^66^, ^67^ ^]^ Indeed, when a flammability test was carried out with ZGPE‐5 using a combustion tool, the gel electrolyte neither caught fire nor shrank confirming its good thermal stability (Figure S25, Supporting Information). Furthermore, considering the usage environment of wearable devices, which transforms according to body movements, the power source for wearable devices should retain its performance under mechanical deformation. Hence, a Zn|ZVO pouch cell was assembled with ZGPE‐5 as an electrolyte, and its electrochemical performance was assessed under various mechanical deformation conditions (Figure 5f). Upon comparing the changes in normalized capacity with respect to the battery capacity in the pristine state, it is found that the mechanical robustness of ZGPE‐5 and the stabilization of Zn deposition ensure stable maintenance of the initial capacity even under 90° bent and 180° folding conditions. As a proof of concept, two cells employing ZGPE‐5 were connected in series to a red LED bulb, demonstrating the capability to consistently light the bulb in flat, 90°, and 180° folded states, thereby proving its potential for application in wearable devices (Figure 5g). This test result proves the potential feasibility of ZGPE‐5 for wearable Zn metal batteries owing to its mechanical flexibility and superior anode reversibility that enables prolonged cyclability.

## 3 Conclusion

In conclusion, we examined the regulated interaction between a zwitterionic polymer matrix and dissolved Zn salt to enhance the anode reversibility and prolong the cell cyclability. Detailed 2D COS analysis reveals that the coordination priority order of functional groups within the zwitterionic unit changes as the ZnSO_4_ concentration increases from 2 to 5 m. The modified priority order encourages the preferential coordination of the polar sulfonate group, thereby intensifying interactions with the Zn^2+^ ions and alleviating the internal concentration gradient. Spectroscopy and electrochemical analysis performed using ZGPE of different concentrations demonstrated that the electrodes employing ZGPE‐2 display vertically grown Zn crystals, whereas cells using ZGPE‐5 show a uniformly grown and dendrite‐free anode surface due to preferential coordination. Thus, ZGPE‐5 exhibited a cycle life exceeding 3600 h in a Zn|Zn symmetric cell test with anode reversibility notably improved compared to ZGPE‐2. Taking advantage of ZGPE‐5, Zn|ZVO full cell using ZGPE‐5 maintained stable working performance beyond 600 cycles, retaining ≈85% of the initial capacity. Briefly, this work illuminates the modification in the interaction between polymer chains and salt within the zwitterionic gel electrolyte with changes in electrolyte concentration, offering fresh insight into the design of advanced electrolytes for AZMBs.

## 4 Experimental Section

### Fabrication of ZGPE

ZGPE was fabricated by redox polymerization of the precursor mixture. In detail, 2.75 g of sulfobetaine methacrylate (10 mmol, SBMA), 1 wt.% of poly(vinyl alcohol) in relation to SBMA, 50 µL of poly(ethylene glycol) diacrylate (0.05 mmol, PEGDA *M*_n_ 250) as a cross‐linker, 20 µL of tetramethylethylenediamine (0.05 mmol, TEMED), and 6 mg of ammonium persulfate (0.05 mmol, APS) were added to 5 mL of distilled water and stirred overnight to fully dissolve precursor materials. The pre‐gel solution was transferred to a silicon mold with 500 µm thickness and polymerized at 4 °C for 12 h to obtain zwitterionic hydrogel. The obtained hydrogel was subsequently immersed into the aqueous solutions containing 2 and 5 m of ZnSO_4_ to prepare ZGPE‐2 and ZGPE‐5, respectively. All chemical reagents were purchased from Sigma–Aldrich and used without additional purification steps.

### Synthesis of Zn_0.25_V_2_O_5_ Powders

Zn_0.25_V_2_O_5_ (ZVO) powder was prepared according to a previous study.^[^ ^65^ ^]^ Briefly, 2.8 mmol of vanadium(V) oxide (Sigma–Aldrich) was dissolved in 29.4 mL of distilled water and vigorously stirred for an hour. The solution was transferred to the oil bath and heated at 40 °C with continuous stirring. Subsequently, 7 mL of hydrogen peroxide aqueous solution (34.5 wt.%; Samchun Chemicals) was slowly added dropwise to the as‐prepared vanadium oxide solution and the mixture was kept stirring for 30 min. Subsequently, 0.7 mmol of zinc acetate dihydrate (Sigma—Aldrich) was dissolved into the above solution and stirred further for 30 min under heating to form a homogeneous precursor solution. Afterward, the solution was moved to a Teflon‐lined autoclave and heated in an oven at 215 °C for 48 h.

### Material Characterization

The chemical composition and ion coordination behavior was estimated using Fourier transform infrared spectroscopy (Shimadzu IRXross). The mechanical properties of fabricated gel electrolytes were measured by a universal tensile machine (Petrol LAB DA‐01) at a constant strain rate of 10 mm min^−1^, where samples were prepared with dimensions of 3 cm × 1 cm with a thickness of 1 mm. X‐ray diffraction (Rigaku D/MAX‐2500/PC) with Cu Kα radiation (*λ* = 1.5406 Å) was utilized to analyze the crystal structure of ZVO and electrode surface degradation. Images of the overall morphology of materials and structural changes in Zn anodes were collected using a field‐emission scanning electron microscope (Hitachi S‐4800). The crystalline structure and nano‐scale configuration of cathode material were investigated using a field emission transmission electron microscope (Jeol JEM‐2100F) under an acceleration voltage of 200 kV. Confocal images were recorded using a confocal laser scanning microscopy (Leica STELLARIS 5 CLSM) equipped with a UV laser with a wavelength of 405 nm and a lens operating in a fluorescence mode. Before the investigation, the hydrogels were immersed in 0.005 wt.% of 8‐anilino‐1‐naphthalenesulfonic acid aqueous solution for 2 days at room temperature. A 3D view was constructed from sliced 2D images using LAS AF software.

### Electrolyte Retention Measurements

Hydrogels (pristine hydrogel, ZGPE‐2, and ZGPE‐5) with a diameter of 12 mm and a thickness of 0.5 mm were prepared to determine the electrolyte retention rate. Subsequently, hydrogels with different ZnSO_4_ contents were placed in a humid chamber (Changshin Science C‐CTHC), and the changes in weight of the samples over time were recorded at 25 °C for 12 h with a relative humidity of 40%, 60%, and 80%. The electrolyte retention rate (W_r_) was calculated by the following equation:

(1) $$\;W_{r}=\frac{W_{t}}{W_{0}}\times 100$$

where *W*_t_ represents the instantaneous weight of the hydrogels at a given time, and *W*_0_ corresponds to the initial weight of the hydrogels.

### Raman 2D Correlation Spectroscopy Investigation

Raman spectra were obtained using a Raman spectrometer (NOST FEX‐MD) in the range of 300—4000 cm^−1^. The ZGPE samples with ZnSO_4_ concentrations ranging from 0 to 5 m were placed on glass slides and analyzed, where a laser with a wavelength of 532 nm was employed to excite the samples under a laser power of 30 mW for respective measurement. The acquisition time for a single measurement was set at 10 s. Following this, the 2D Shige program was used for 2D COS measurements, yielding both synchronous and asynchronous spectra results. Before carrying out 2D gradient mapping, Raman spectra results were subjected to baseline correction and intensity normalization.

### Electrochemical Measurements

Electrochemical performances were estimated using 2032‐type coin cells, where zinc metal was adopted as both a counter electrode and a reference electrode. For symmetric cell tests, the stabilizing step was conducted and cells were operated under 0.5 mA cm^−2^ for 10 cycles with an areal capacity of 0.5 mAh cm^−2^. The cathode slurry was prepared by uniformly mixing ZVO powders as an active material, carbon black as a conducing agent, and polyvinylidene fluoride as a binder in N‐methyl‐2‐pyrrolidone at a weight ratio of 7:2:1. Subsequently, the as‐prepared slurry was blade coated on titanium foil current collector and vacuum dried at 70 °C for 20 h. The dried electrode was punched into disks with a diameter of 12 mm. The loading mass of the cathode active materials was 1.8—2 mg cm^−2^. The galvanostatic charge‐discharge investigation of Zn|Zn symmetric cells and Zn|ZVO full cells was conducted using a battery cycler system (Wonatech WBCS3000Le). The EIS, cyclic voltammetry, and chronoamperometry analysis were tested by an electrochemical workstation (Bio‐logic Science Instruments VMP‐300). To measure the internal resistance of cells, EIS analysis was carried out in the frequency range from 1 MHz to 10 mHz with an applied amplitude of 10 mV.

## Conflict of Interest

The authors declare no conflict of interest.

## Supporting information

Supporting Information

## Data Availability

The data that support the findings of this study are available from the corresponding author upon reasonable request.
