# Supplementary material for: Promoting Homogeneous Zinc‐Ion Transfer Through Preferential Ion Coordination Effect in Gel Electrolyte for Stable Zinc Metal Batteries
Source: Adv Sci (Weinh). 2023 Oct 23;10(34):2304915. doi: 10.1002/advs.202304915 (PMC10700204; doi:10.1002/advs.202304915)
Supplement: Supplementary file 1 — Supporting Information [file ADVS-10-2304915-s001.pdf]

ADVANCED SCIENCE Open Access

Supporting Information

for *Adv. Sci.*, DOI 10.1002/advs.202304915

Promoting Homogeneous Zinc-Ion Transfer Through Preferential Ion Coordination Effect in Gel Electrolyte for Stable Zinc Metal Batteries

Sangyeop Lee, Im Kyung Han, Na Gyeong Jeon, Yubin Lee, Hye Bin Son, Dong-Yeob Han, Seoha Nam, Taehun Chung, Myung-Jun Kwak, Youn Soo Kim and Soojin Park**

Supporting Information

Promoting Homogeneous Zinc-ion Transfer through Preferential Ion Coordination Effect in Gel Electrolyte for Stable Zinc Metal Batteries

Sangyeop Lee, Im Kyung Han, Na Gyeong Jeon, Yubin Lee, Hye Bin Son, Dong-Yeob Han, Seoha Nam, Taehun Chung, Myung-Jun Kwak, Youn Soo Kim, and Soojin Park**

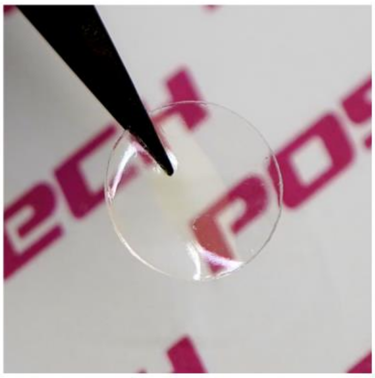

Figure S1. Digital microscope image displaying the fabricated ZGPE after being cut into a disk with a diameter of 19 mm.

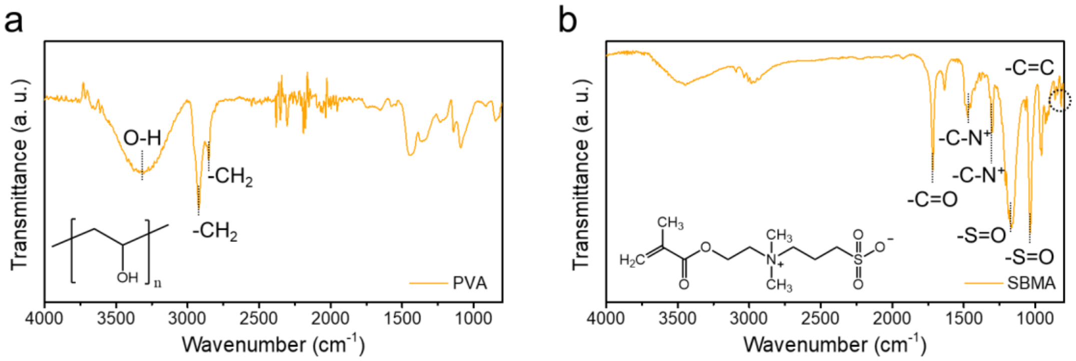

Figure S2. FTIR spectra of a) PVA polymer and b) SBMA repeating unit. Inset images show representative molecular structure of PVA and SBMA.

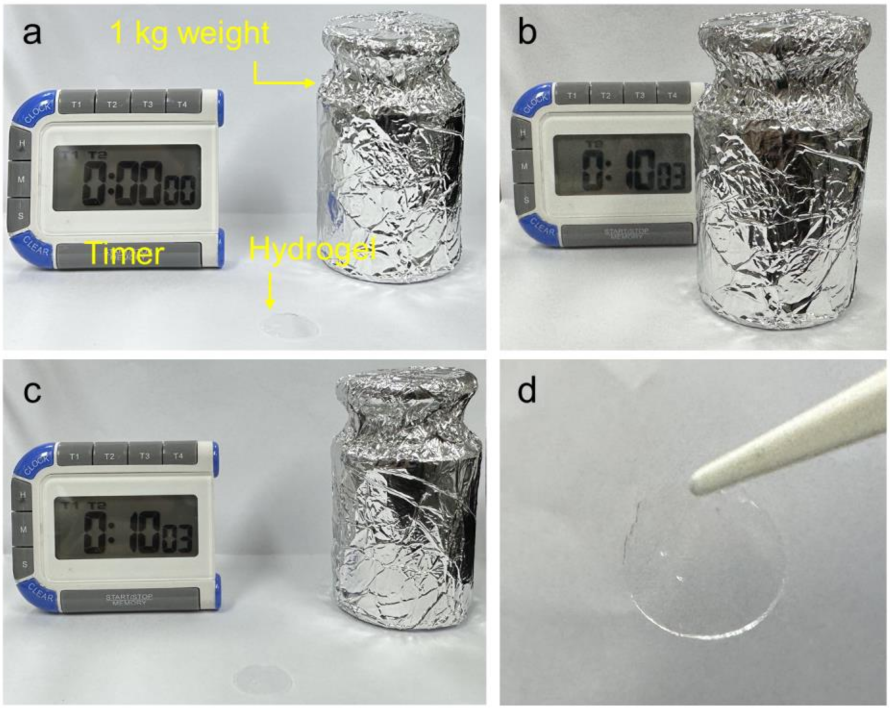

Figure S3. Mechanical stability investigation of ZGPE. Digital microscope captures depict a) ZGPE prior to the imposition of a 1 kg load, b) ZGPE subjected to compressive stress for 10 minutes, and c) ZGPE after load removal. d) A magnified image of ZGPE following the durability, revealing an absence of notable fractures.

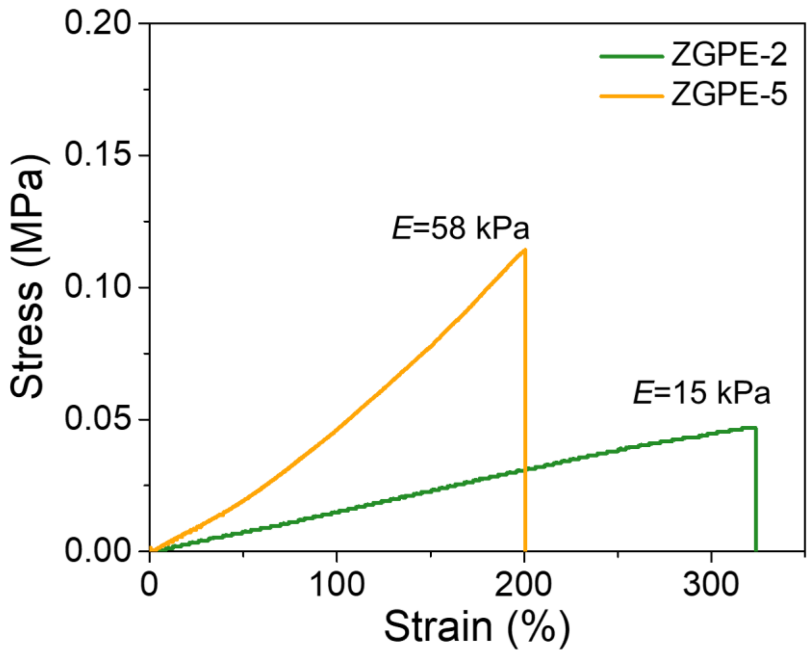

Figure S4. Stress-strain curves of the ZGPE-2 and the ZGPE-5 gels under a constant elongation rate of 10 mm·s^{-1}. Young's modulus (*E*) values for the ZGPE-2 and the ZGPE-5 are 15 kPa and 58 kPa respectively.

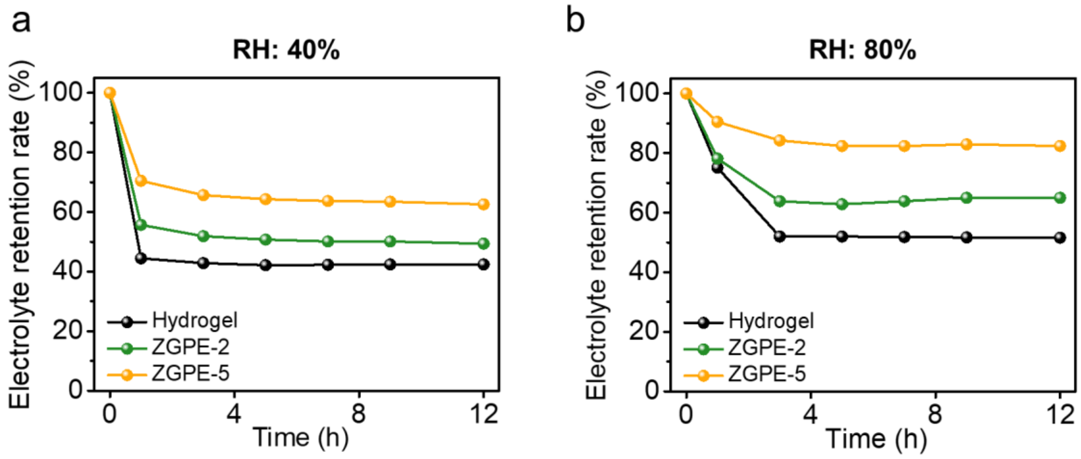

Figure S5. Electrolyte retention rate of the pristine hydrogel, ZGPE-2, and ZGPE-5 over time at room temperature under a relative humidity (RH) of 40 and 80%, respectively.

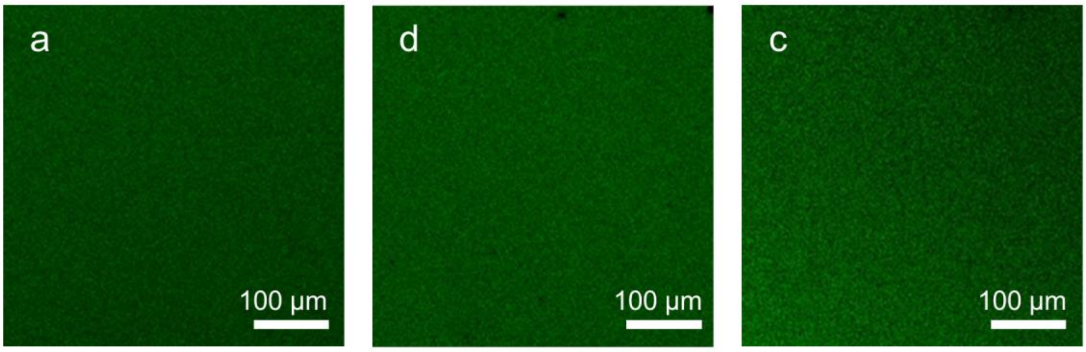

Figure S6. Three-dimensional confocal laser scanning microscopy images of a) pristine hydrogel, b) ZGPE-2, and c) ZGPE-5 at room temperature.

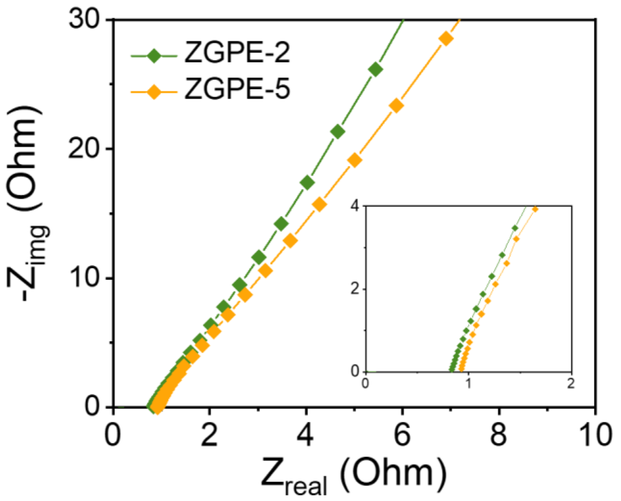

Figure S7. Electrochemical impedance spectroscopy investigation of ZGPE-2 and ZGPE-5 at room temperature. The ionic conductivity (σ), was calculated using the following equation:

$$\sigma = \frac{l}{\mathrm{R_b} A}$$

, where R_b is the bulk resistance, and l and A are the thickness and area of the gel electrolyte, respectively.

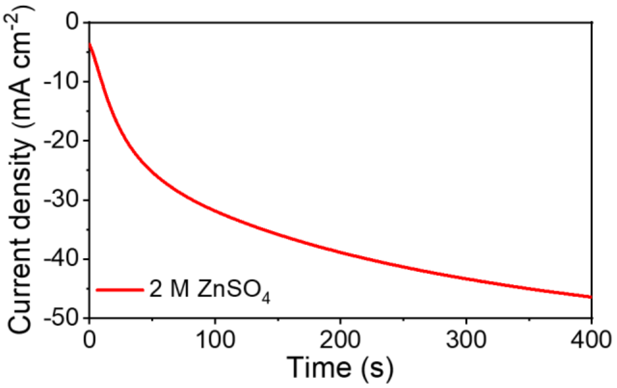

Figure S8. Time-current density plot of a cell employing 2 M $ZnSO_4$ liquid electrolyte for 400 seconds.

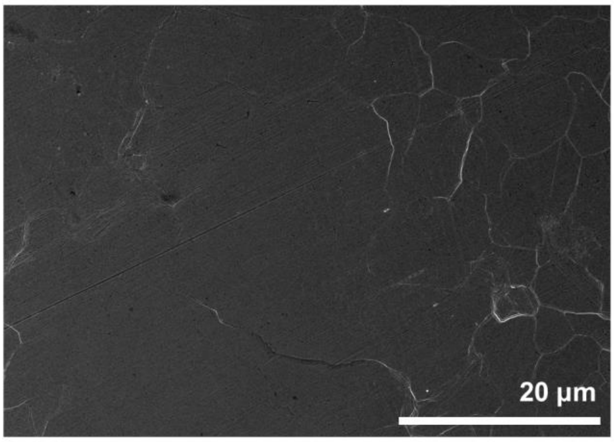

Figure S9. Top-view SEM image of polished Zn foil.

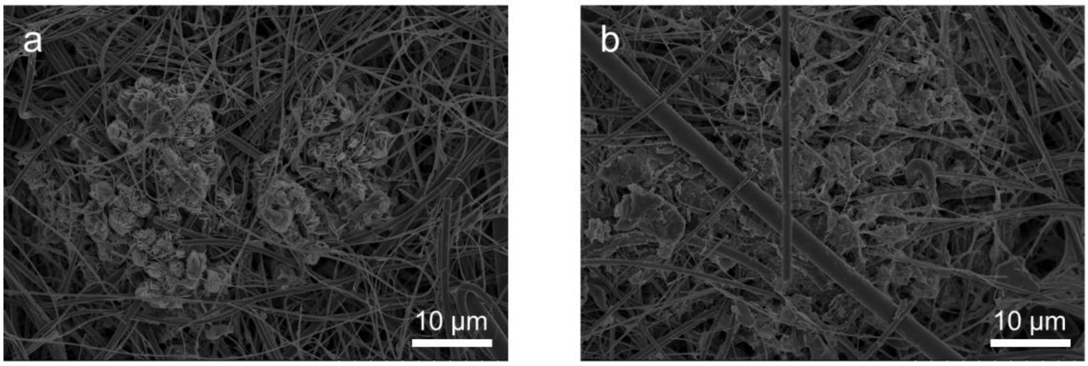

Figure S10. Top-view SEM image of Zn anodes after plating 3 mAh·cm^{-2} of Zn using a) 2 m $ZnSO_4$ and b) 5 m $ZnSO_4$ liquid electrolyte. Current density is 0.5 mA·cm^{-2}.

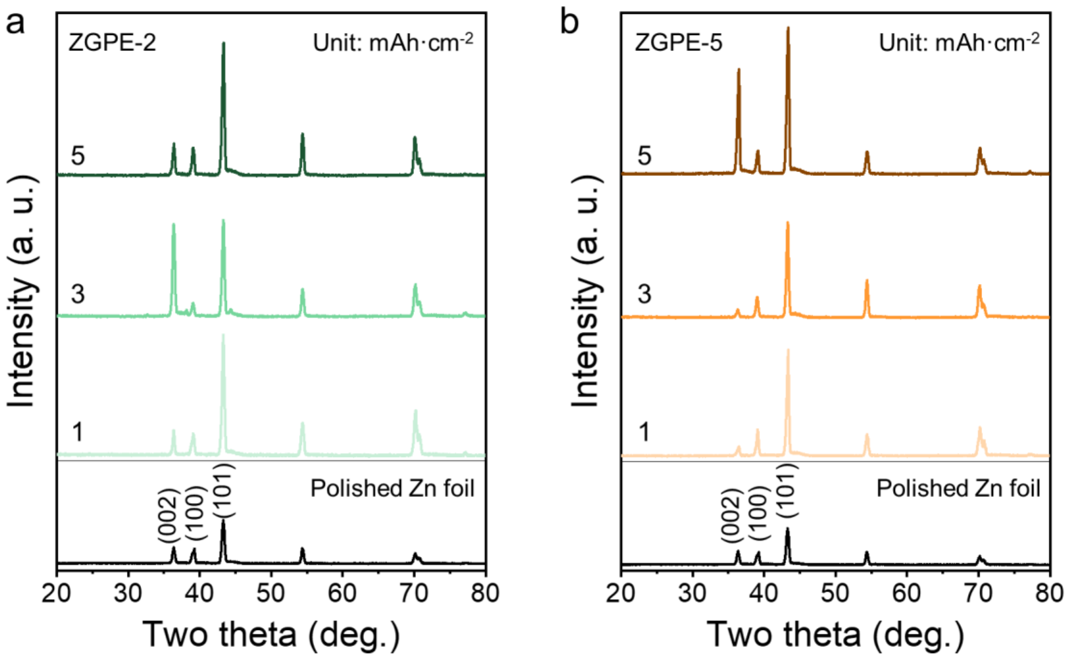

Figure S11. XRD analysis results of Zn foil after depositing 1, 3, and 5 mAh·cm^{-2} of additional Zn under a current density of 0.5 mA·cm^{-2} employing a) ZGPE-2 and b) ZGPE-5 as an electrolyte.

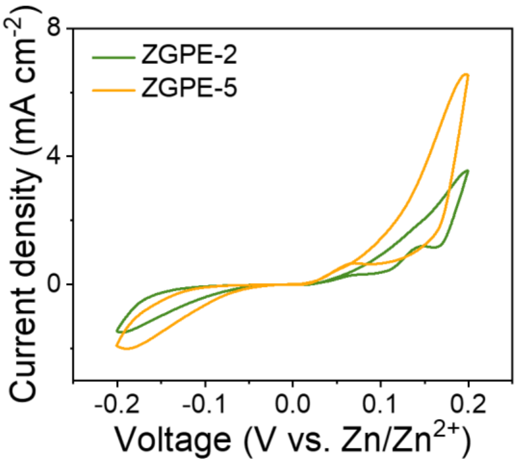

Figure S12. Cyclic voltammetry curves for cells employing ZGPE-2 and ZGPE-5. The scan rate is 1 $mV{\cdot}s^{-1}$.

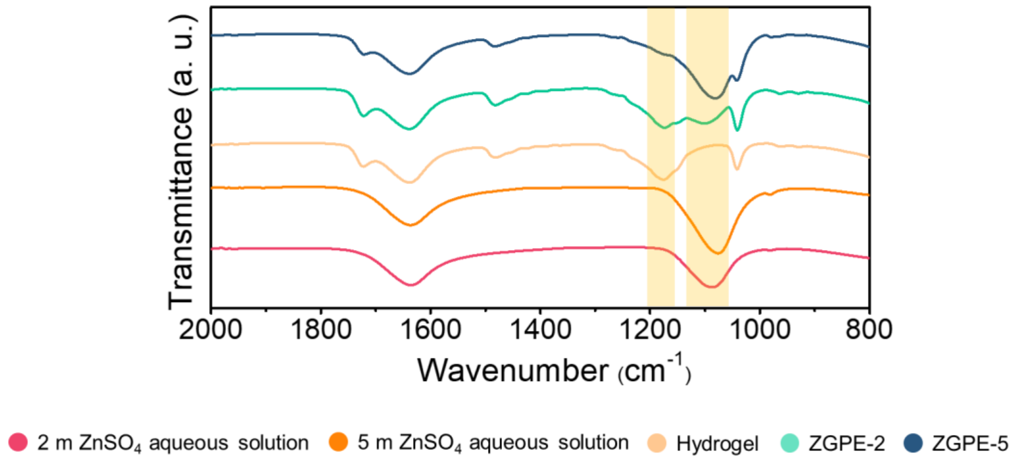

Figure S13. FTIR spectra of $ZnSO_4$ aqueous solution and ZGPE with various salt concentration condition. Peaks at 1030 cm^{-1} and 1170 cm^{-1} represent characteristic peaks of S=O vibrations coming from sulfonate functional groups in SBMA, while peak at 1085 cm^{-1} is attributed to the sulfate contained in $ZnSO_4$. For the peak at 1085 cm^{-1}, red shift occurs when the electrolyte concentration increases from 2 m to 5 m due to the intensified interaction between S=O and Zn^{2+} ions. Additionally, same peak at ZGPE undergoes blue shift compared with liquid electrolyte as Zn^{2+} ions favorably interact with the gel matrix, enhancing the bond strength of S=O. Meanwhile, the intensity of peak at 1170 cm^{-1} decreases with increment of concentration because robust interaction between ZGPE structure and internal Zn^{2+} ions hinders asymmetrical vibration of S=O in zwitterionic polymer chains.

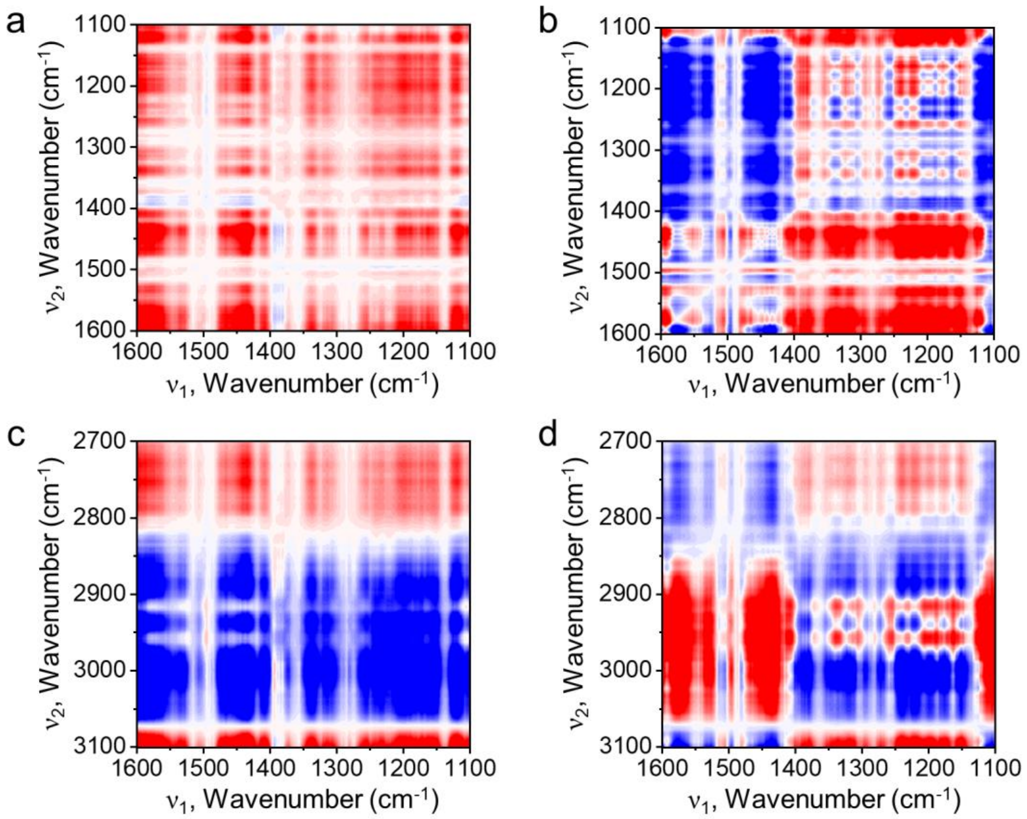

Figure S14. a, c) Synchronous and b, d) asynchronous two-dimensional Raman correlation spectra (2DCOS) of ZGPE during increasing salt concentration 0 m to 2 m.

Slow ⟶ **Fast**

0 m → 2 m			
1449 cm^{-1}	1123 cm^{-1}	2940 cm^{-1}	1329 cm^{-1}
$-CH_2$	SO_3^-	$-N^+(CH_3)_2$	O-C=O

Figure S15. The sequential order of spectral changes of ZGPE as revealed by Raman analysis with increasing salt concentration from 0 m to 2 m.

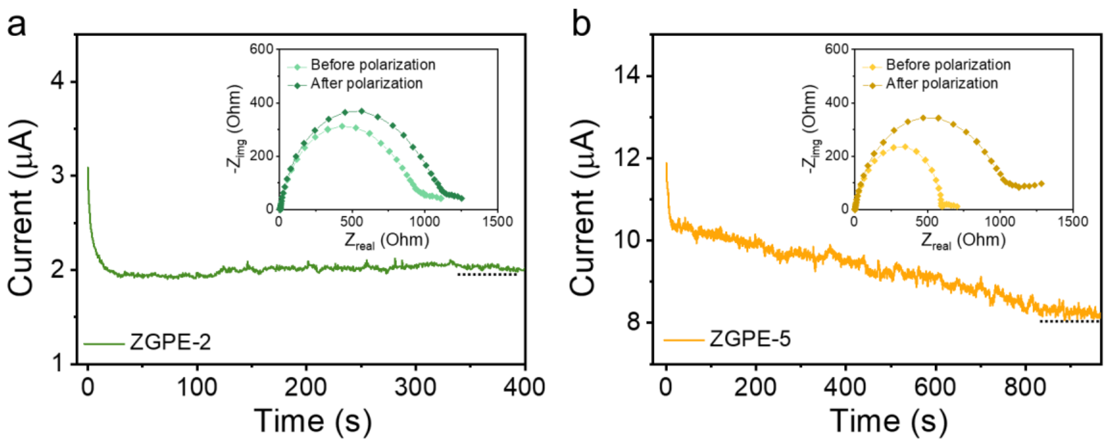

Figure S16. Current-time plots of cells employing a) ZGPE-2 and b) ZGPE-5 as an electrolyte. The inset figure shows EIS results of respective cells before and after polarization. The applied voltage polarization is 10 mV at room temperature.

The transference number of Zn^{2+} (t_{Zn2+}) was calculated using the following equation:

$$t_{Zn^{2+}} = \frac{I_s(\Delta V - I_0 R_0)}{I_0(\Delta V - I_s R_s)}$$

where ΔV is the applied voltage polarization, I_0 and I_s are the initial state current and steady-state current, respectively, and R_0 and R_s are the initial state resistance and steady-state resistance, respectively.

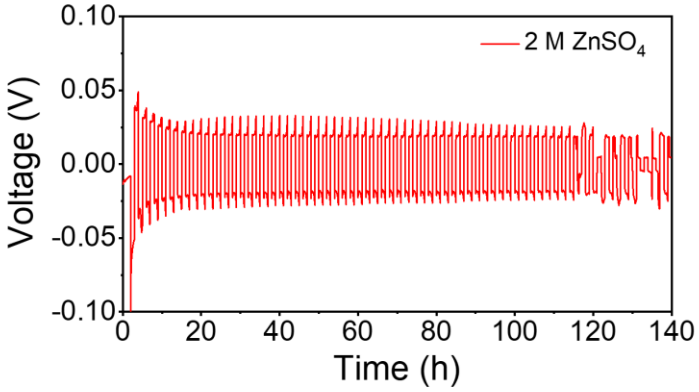

Figure S17. Symmetric cell cycling result using 2 M $ZnSO_4$ liquid electrolyte at an areal capacity of 0.5 mAh·cm^{-2} under a current density of 0.5 mA·cm^{-2}.

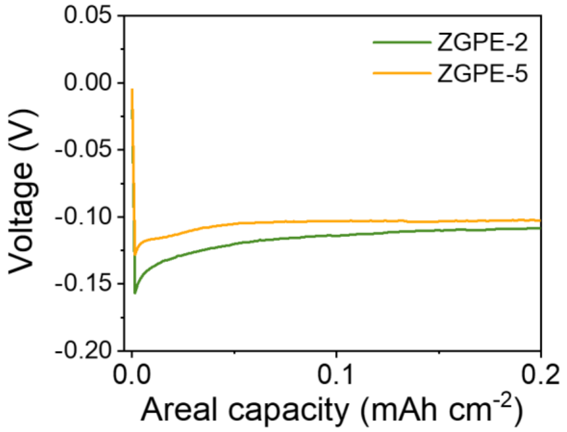

Figure S18. The voltage-areal capacity curves during initial Zn electrodeposition process at 0.5 mA cm^{-2} for Zn|Zn symmetric cells employing ZGPE-2 and ZGPE-5, respectively. The inset demonstrates an enlarged view of the area marked by the red dashed line in voltage-areal capacity curves.

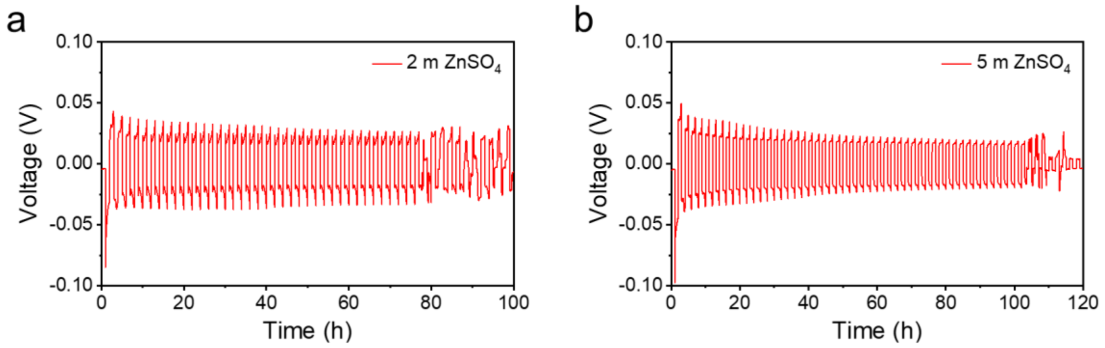

Figure S19. Symmetric cell cycling results using a) 2 m $ZnSO_4$ and b) 5 m $ZnSO_4$ liquid electrolyte at an areal capacity of 0.5 mAh·cm^{-2} under a current density of 0.5 mA·cm^{-2}.

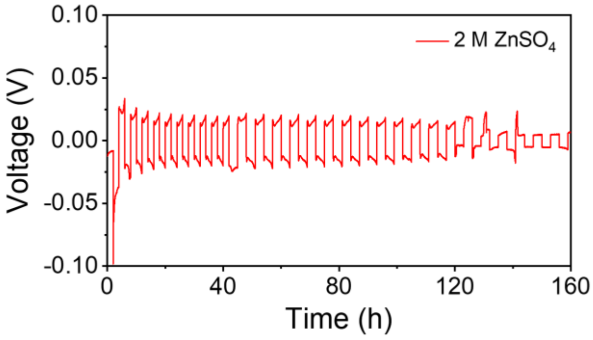

Figure S20. Symmetric cell cycling result using 2 M $ZnSO_4$ liquid electrolyte at an areal capacity of 3 mAh·cm^{-2} under a current density of 1 mA·cm^{-2}.

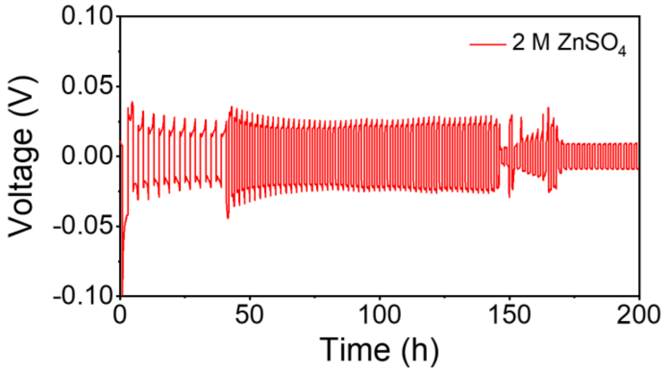

Figure S21. Symmetric cell cycling result using 2 M $ZnSO_4$ liquid electrolyte at an areal capacity of 2 mAh·cm^{-2} under a current density of 2 mA·cm^{-2}.

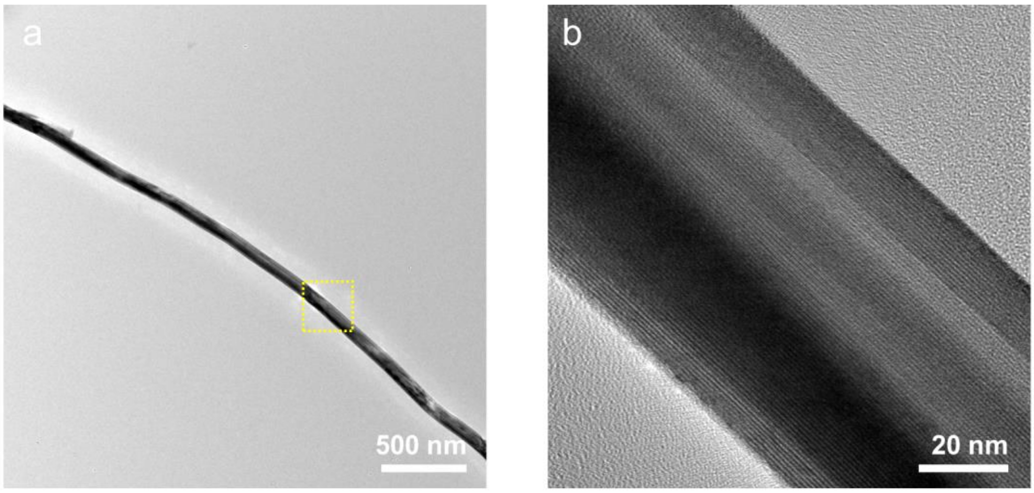

Figure S22. Transmission electron microscope images of $Zn_{0.25}V_2O_5$ cathode active material for a) overall morphology and b) detailed image demonstrating highly ordered crystal structure.

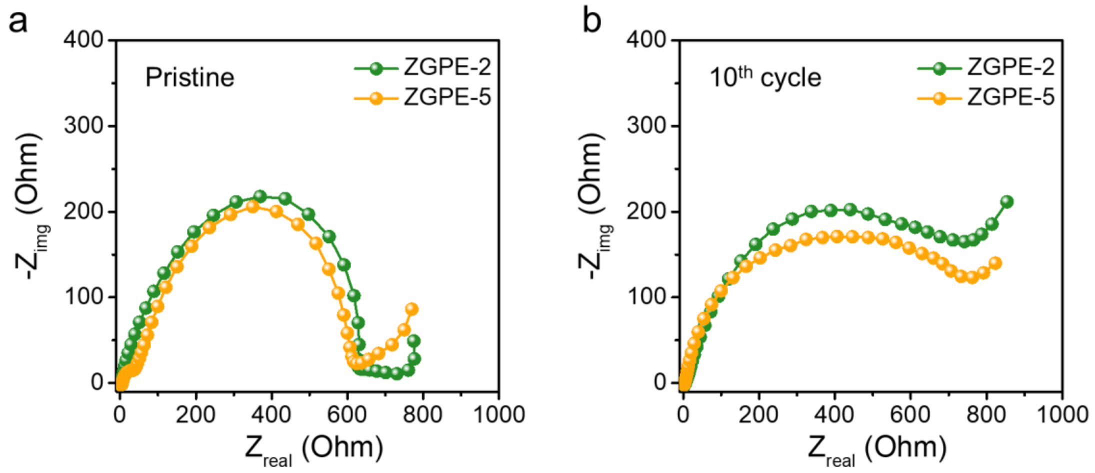

Figure S23. Nyquist plots associated with Zn|ZVO full cells utilizing ZGPE-2 and ZGPE-5 as respective electrolytes, assessed a) prior to cell operation and b) after 10th cycle.

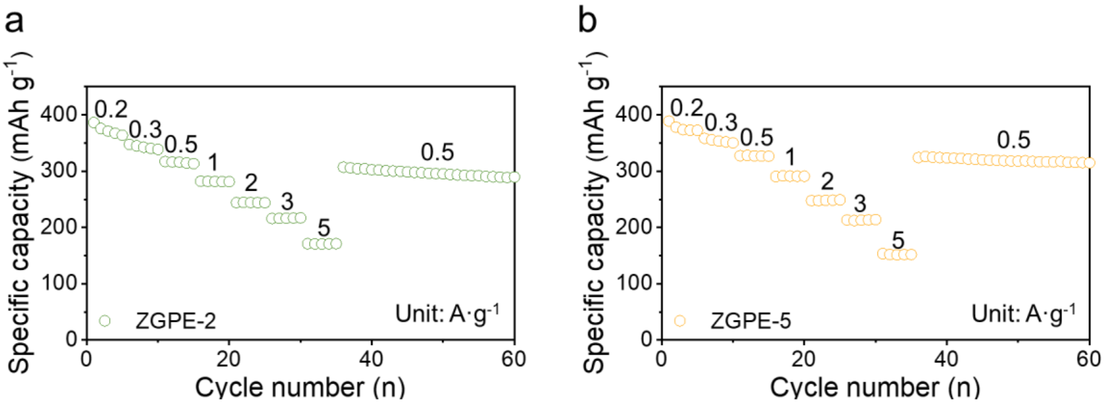

Figure S24. Rate performance of the a) ZGPE-2 and b) ZGPE-5 based Zn|ZVO full cells.

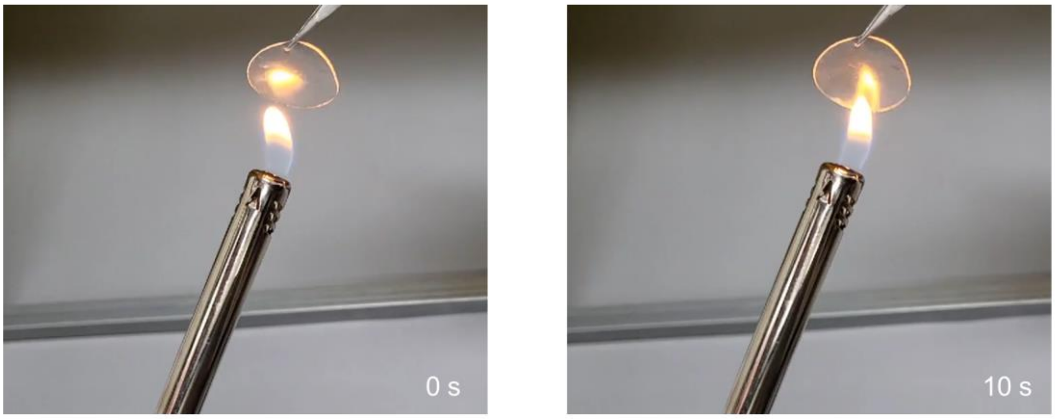

Figure S25. Digital microscope images of ZGPE-5 during flammability test, with the gel electrolyte directly subjected to flame via a combustion tool. (left) Onset of the test and (right) following 10 seconds of exposure to an ignition condition.

Table S1. Summary of the Raman investigation for respective peak position and corresponding synchronous/asynchronous results with increasing salt concentration from 0 m to 2 m.

Peak position	Synchronous	Asynchronous
1123, 1329	Positive	Negative
1123, 1449	Positive	Positive
1329, 1449	Positive	Positive
1123, 2940	Negative	Positive
1329, 2940	Negative	Negative
1449, 2940	Negative	Positive

Table S2. Summary of the Raman investigation for respective peak position and corresponding synchronous/asynchronous results with increasing salt concentration from 0 m to 5 m.

Peak position	Synchronous	Asynchronous
1123, 1329	Positive	Negative
1123, 1449	Positive	Positive
1329, 1449	Positive	Positive
1123, 2940	Negative	Positive
1329, 2940	Negative	Negative
1449, 2940	Negative	Positive

Table S3. The summary of various zinc metal batteries using hydrogel electrolytes based on recently reported literatures.

Gel Matrix	Salt	Areal Capacity ($mAh \cdot cm^{-2}$)	Life time (h)	Cumulative Capacity ($mAh \cdot cm^{-2}$)	Ref.
SBMA + PVA + PEGDA	5 m $ZnSO_4$	0.5	3600	900	This work
PEGDM + PP	2 M $Zn(ClO_4)_2$	0.5	1000	250	S1
Gum Arabic	1 M $ZnSO_4$ + 1 M Li_2SO_4	0.2	1400	140	S2
PVA + TiO_2	$Zn(CF_3SO_3)_2$	0.5	3000	750	S3
PAM + Glycerol + Acetonitrile	3 M $ZnSO_4$	5	3000	750	S4
PVA + Xanthan Gum	2 M $ZnSO_4$	0.1	2000	100	S5
PEO + Cyclodextrin	2 M $ZnSO_4$	0.7	1400	490	S6
DMAPS + AM	2 M $Zn(ClO_4)_2$	0.5	3000	750	S7
Wool Keratin + Carrageenan	1 M $ZnSO_4$	0.5	2000	500	S8

References

[S1] X. Lin, G. Zhou, J. Niu, M. J. Robson, J. Yu, Y. Wang, Z. Zhang, S. C. T. Kwok, F. Ciucci, *Adv. Funct. Mater.* **2021**, *31*, 2105717.

[S2] K. Wu, J. Cui, J. Yi, X. Liu, F. Ning, Y. Liu, J. Zhang, *ACS Appl. Mater. Interfaces* **2022**, *14*, 34612.

[S3] C. Liu, Y. Tian, Y. An, Q. Yang, S. Xiong, J. Feng, Y. Qian, *Chem. Eng. J.* **2022**, *430*, 132748.

[S4] T. Wei, Y. Ren, Z. Li, X. Zhang, D. Ji, L. Hu, *Chem. Eng. J.* **2022**, *434*, 134646.

[S5] C. Fu, Y. Wang, C. Lu, S. Zhou, Q. He, Y. Hu, M. Feng, Y. Wan, J. Lin, Y. Zhang, A. Pan, *Energy Storage Mater.* **2022**, *51*, 588.

[S6] K. Wu, S. Zhan, W. Liu, X. Liu, F. Ning, Y. Liu, J. Zhang, J. Yi, *ACS Appl. Mater. Interfaces* **2023**, *15*, 6839.

[S7] Q. He, G. Fang, Z. Chang, Y. Zhang, S. Zhou, M. Zhou, S. Chai, Y. Zhong, G. Cao, S. Liang, A. Pan, *Nano-Micro Lett.* **2022**, *14*, 93.

[S8] Y. Shao, J. Zhao, W. Hu, Z. Xia, J. Luo, Y. Zhou, L. Zhang, X. Yang, N. Ma, D. Yang, Q. Shi, J. Sun, L. Zhang, J. Hui, Y. Shao, *Small* **2022**, *18*, 2107163.
